# Association between suicide, external-cause and all-cause mortality and irregular mental health discharge among the US veteran population

**DOI:** 10.1192/bjo.2021.1000

**Published:** 2021-09-02

**Authors:** Natalie B. Riblet, Daniel J. Gottlieb, Bradley V. Watts, Maxwell Levis, Brian Shiner

**Affiliations:** Veterans Affairs Medical Center, USA; and Geisel School of Medicine at Dartmouth College, USA; Veterans Affairs Medical Center, USA; Veterans Affairs Medical Center, USA; Geisel School of Medicine at Dartmouth College, USA; and VA Office of Systems Redesign and Improvement, USA; Veterans Affairs Medical Center, USA; and Geisel School of Medicine at Dartmouth College, USA; Veterans Affairs Medical Center, USA; Geisel School of Medicine at Dartmouth College, USA; and National Center for PTSD, USA

**Keywords:** Deliberate self-harm, in-patient treatment, mortality, rehabilitation, suicide

## Abstract

**Background:**

Irregular hospital discharge is highly prevalent among people admitted to hospital for mental health reasons. No study has examined the relationship between irregular discharge, post-discharge mortality and treatment setting (i.e. mortality after patients are discharged from acute in-patient or residential mental health settings).

**Aims:**

To understand the relationship between irregular discharge and mortality among patients discharged from acute in-patient and residential settings.

**Method:**

A retrospective study was conducted in members of the US veteran population discharged from acute in-patient or residential settings of the US Department of Veterans Affairs between 2003 and 2018. Multivariate Cox proportional hazards were used to evaluate associations between irregular discharge and suicide, external-cause (as defined by ICD-10 Codes: V01-Y98) and all-cause mortality in the first 30-, 90- and 180-days post-discharge.

**Results:**

There were over 1.5 million mental health discharges between 2003 and 2018. Patients with an irregular discharge were at increased risk for suicide, external-cause and all-cause mortality in the first 180 days after discharge. In the first 30 days after discharge, patients with irregular discharge had more than three times greater suicide risk than patients with regular discharge (adjusted hazard ratio (HR) = 3.41, 95% CI 2.21–5.25). Suicide risk was higher among patients with irregular discharge in the first 30 days after acute in-patient discharge (adjusted HR = 1.55, 95% CI 1.11–2.16). In both settings, the mortality risk associated with irregular discharge attenuated but remained elevated within 90 and 180 days.

**Conclusions:**

Irregular discharge after an acute in-patient or residential stay poses a large risk for mortality soon after discharge. Clinicians must identify effective interventions to mitigate harms associated with irregular discharge in these settings.

Health system and patient factors play a critical role in health outcomes during care transitions.^1^ Some of the strongest predictors of negative health outcomes in the post-hospital discharge period include poorly coordinated care transitions as well as a lack of patient engagement in treatment.^1–3^ Among mental health populations, irregular discharges, including discharge against medical advice and other unplanned discharges such as self-initiated discharge, are of special concern.^2–4^ Research has shown that irregular discharge is common among patients with mental health disorders and substance use disorders (SUDs), with rates of irregular discharge reported as high as 50% among those admitted with mental health conditions.^5^ It is particularly worrisome that irregular discharge rates continue to rise among patients with SUDs or substance-induced mental disorders.^6^

## Suicide mortality and irregular mental health discharge

Evidence suggests that irregular discharge contributes to post-discharge suicide risk.^2,3^ For example, in a population-based cohort study of 5 million psychiatric and general medical discharges from the US Department of Veterans Affairs, Riblet et al observed that patients with irregular discharges were at double the risk for dying by suicide in the first year after discharge when compared with patients with a regular discharge.^2^ After stratification by unit type (medical versus mental health discharge), however, they found that the higher risk for suicide was only evident among patients leaving general medical units. Importantly, this study^2^ as well as a similar study by Kuo et al^3^ did not examine the association between irregular discharge and suicide outcomes across mental health treatment settings (acute in-patient versus residential). The treatment paradigm of each of these settings is distinct. Whereas acute stays provide patients crisis stabilisation,^7^ residential stays provide patients with longer-term behavioural health treatment in a supervised, non-hospital setting.^8^ Given these differences, it is likely that patients who leave an acute in-patient mental health stay may face different risks for suicide mortality in the post-discharge period compared with patients who leave a residential mental health stay.

## Post-discharge temporal trends in suicide mortality and irregular mental health discharge

It is well-known that patients are at high risk for suicide within the first year after acute in-patient mental health discharge, with the risk being the greatest in the first month following discharge.^9,10^ For example, in a pooled analysis of 100 studies reporting on post-discharge suicide risk, Chung et al determined that the rate of suicide per 100 000 person-years in the first 3 months after discharge was 1132 suicides.^10^ This rate fell to 654 suicides between 3 months and 1 year after discharge.^10^ In a separate meta-analysis of 29 studies, Chung et al also observed that the rates of suicide per 100 000 person-years in the first month after discharge far exceeded those reported at 3 months (2060 suicides *v.* 1132).^9,10^ To the best of our knowledge, no study has considered how an irregular discharge may be related to these temporal trends.

## Study objectives

To address current knowledge gaps, we aimed to understand the relationship between irregular discharge and mortality among members of the US veteran population discharged from acute in-patient or residential mental health treatment settings within the Veterans Affairs healthcare system. We hypothesised that patients with an irregular discharge would be at greater risk for death by suicide. We also hypothesised that the risk for suicide would be the highest in the first 30 days after discharge and would then diminish. We included external-cause and all-cause mortality as secondary outcomes because people with mental health and SUDs are at higher risk of these deaths.^11^ A better understanding of how irregular discharge has an impact on mortality risk following treatment in acute in-patient and residential mental health settings will contribute to the design of more tailored interventions to decrease suicide risk in the period after leaving hospital.

## Method

### Study design

We carried out a retrospective cohort study of all patients discharged from Veterans Affairs acute in-patient or residential mental health treatment settings (herein after referred to as acute in-patient and residential) between 1 January 2003 and 31 December 2018. Our cohort included veterans of US military service. Currently more than 90% of US veterans are men.^12^ Because active conscription ended in the USA in 1973, our cohort largely comprises people who volunteered for service. A small population of non-veterans use Veterans Affairs healthcare, including active-duty service members, former service members with other than honourable discharges, as well as family members of veterans who are severely disabled who are eligible for Veterans Affairs healthcare. All Veterans Affairs healthcare users, the vast majority of whom are US veterans, were eligible for inclusion in our cohort.

We used discharge disposition codes to identify regular (value, 1) and irregular discharges (value, 4). We included all patients who were discharged to the out-patient setting. We excluded patients whose discharge type code indicated that the patient died in the hospital or who were transferred to another in-patient setting at discharge. We also excluded admissions with a primary diagnosis of dementia or non-mental health conditions. We followed patients from the day of discharge for up to 6 months (180 days), with censorship for readmission to a Veterans Affairs mental health treatment setting, maximum follow-up achieved or end of the study period (31 December 2018). A patient could have multiple observations.

The authors assert that all procedures contributing to this work comply with the ethical standards of the relevant national and institutional committees on human experimentation and with the Helsinki Declaration of 1975, as revised in 2008. All procedures involving human patients were approved by the Veteran's institutional review board of Northern New England (USA), approval number 988703-18. A waiver of consent and authorisation was granted for the study.

### Covariates

We used the following covariates in our models to account for potential confounders: age, gender, ethnicity (Black, Hispanic, White, Other), marital status, comorbidities, homelessness, rurality, primary discharge diagnosis and year of admission. We assessed homelessness in the 2 calendar years prior to the year of admission using a mixture of the International Statistical Classification of Diseases and Related Health Problems (ICD) codes^13,^^14^ as well as clinic codes for use of homelessness-related services.^15^ Age and marital status were assessed at the year of admission. Age was categorised as follows: 18–35, 36–49, 50–59, 60–69 and ≥70 years. For all diagnostic bins, we required a single instance from an in-patient facility or two or more out-patient encounters 7 to 365 days apart. Physical and mental health comorbidities were assessed in the 2 years prior to date of admission using published bins of related ICD-9^14^ and ICD-10 codes.^13^^,^^16^ For mental health comorbidities, we coded the number of Diagnostic and Statistical Manual of Mental Disorders (5th edition) (DSM-5) categories (0–1, 2–3 and ≥4).^17^ For physical health comorbidities, we coded diagnoses based on the number of Elixhauser conditions (0, 1, and ≥2).^18^^,^^19^ For this application, we excluded hypertension without complications. We collapsed diabetes with and without complications as well as different cancer diagnoses each into a single condition. Primary discharge diagnoses were binned as follows: SUDs, alcohol use disorders (AUDs), bipolar disorders, depressive disorders, psychotic disorders, trauma-related disorders and other mental health disorders. We determined zip code of residence annually and relied on the Rural-Urban Commuting Area (RUCA) classification scheme to define RUCA codes 1–3 as urban and all others as rural.^16,20^ Finally, we separated year of admission into four time periods (2003–2007, 2008–2011, 2012–2015 and 2016–2019) in order to adjust for and evaluate temporal trends.

### Outcomes

Our primary outcome of interest was death by suicide (defined as ICD-10: X60-X84, Y87.0, U03). Our secondary outcomes of interest were external causes of mortality (defined as ICD-10: V01-Y98) and all-cause mortality. All outcomes were nested within each other and not mutually exclusive. We identified date of death and cause of death for decedents within our study cohort using the Veterans Affairs Department of Defense Mortality Data Repository. This resource links information on Veterans Affairs decedents to the Centers for Disease Control and Prevention National Death Index.^21^

### Descriptive analysis

In order to characterise the study population, we first performed a descriptive analysis, whereby we stratified the population by treatment setting (acute in-patient and residential) and compared characteristics across strata using chi-squared tests for dichotomous outcomes and independent samples *t*-test for continuous outcomes. We also calculated mortality rates per 100 000 person-years for each setting and reported on the most common external causes of death in the study population.

### Survival analysis

We used a multivariate Cox proportional hazards regression model to evaluate the risks associated with irregular discharge stratified by setting. We stratified by setting because of the large differences in length of stay that would make interpretation of a combined model difficult.^7,8^ We report hazard ratios (HR) with 95% CI.

We ran models censoring patients at 30, 90 and 180 days post-discharge. We adjusted for all covariates as described above. Although patients were censored if they were readmitted to a Veterans Affairs mental health setting, patients continued to contribute data to the risk period if they were admitted to other settings during the follow-up period.

To compare the temporal effects by discharge type and setting, we generated survival curves to visually compare the probabilities of suicide and external-cause mortality within the first 180 days after discharge across the two settings and discharge types.

We conducted data management and analysis using SAS Version 9.4 (SAS Institute, Cary NC).

## Results

### Descriptive analysis

There were over 1.5 million Veterans Affairs mental health discharges between 2003 and 2018. As shown in Table 1, the majority of mental health discharges were from acute in-patient settings (71.0%). There were several important differences between discharges from these two settings. Residential patients were more likely to have an irregular discharge, be of younger age, be of Black ethnicity, and carry a primary discharge diagnosis of SUD or AUD. Homelessness was very common among residential discharges. However, there were no notable differences between settings based on urban–rural classification or year of admission.

**Table 1:** Patient characteristics for total discharges stratified by treatment setting, Department of Veterans Affairs 2003–2018

		Treatment setting	
	Overall	Residential	Acute in-patient
Total discharges, *n* (%)	1 598 567 (100.0)	462 731 (28.9)	1 135 836 (71.0)***
Gender			
Female, *n* (%)	122 364 (7.7)	25 470 (5.5)	96 894 (8.5)***
Male, *n* (%)	1 476 203 (92.3)	437 261 (94.5)	1 038 942 (91.5)***
Age at admission			
Years, mean (s.d.)	49.9 (12.2)	49.0 (11.2)	50.2 (12.6)***
18–35 years, *n* (%)	247 771 (15.5)	71 893 (15.5)	175 878 (15.5)
36–49 years, *n* (%)	442 788 (27.7)	139 134 (30.1)	303 654 (26.7)***
50–59 years, *n* (%)	581 569 (36.4)	175 630 (38.0)	405 939 (35.7)***
60–69 years, *n* (%)	268 643 (16.8)	69 041 (14.9)	199 602 (17.6)***
≥70 years, *n* (%)	57 796 (3.6)	7 033 (1.5)	50 763 (4.5)***
Marital status, *n* (%)			
Never	483 147 (30.2)	136 995 (29.6)	346 152 (30.5)***
Divorced/widowed/separated	739 260 (46.2)	228 188 (49.3)	511 072 (45.0)***
Married	362 195 (22.7)	94 860 (20.5)	267 335 (23.5)***
Unknown	13 965 (0.9)	2 688 (0.6)	11 277 (1.0)***
Ethnicity, *n* (%)			
Black	482 375 (30.2)	162 284 (35.1)	320 091 (28.2)***
Hispanic	89 395 (5.6)	18 990 (4.1)	70 405 (6.2)***
White	977 724 (61.2)	267 245 (57.8)	710 479 (62.6)***
Other	392 929 (2.5)	11 949 (2.6)	27 343 (2.4)***
Unknown	9 781 (0.6)	2 263 (0.5)	7 518 (0.7)***
Primary discharge diagnosis, *n* (%)			
Substance use disorders	268 005 (16.8)	126 331 (27.3)	141 674 (12.5)***
Alcohol use disorders	419 514 (26.2)	202 920 (43.9)	216 594 (19.1)***
Bipolar disorders	134 427 (8.4)	11 075 (2.4)	123 352 (10.9)***
Depressive disorders	283 533 (17.7)	25 493 (5.5)	258 040 (22.7)***
Psychotics disorders	234 877 (14.7)	12 092 (2.6)	222 785 (19.6)***
Trauma-related disorders	226 978 (14.2)	80 479 (17.4)	146 499 (12.9)***
Other mental health disorders	31 233 (2.0)	4 341 (0.9)	26 892 (2.4)***
Pre-existing health conditions, *n* (%)			
Homelessness	612 418 (38.3)	199 551 (43.1)	412 867 (36.3)***
0–1 mental health conditions	283 943 (17.8)	66 887 (14.5)	217 056 (19.1)***
2–3 mental health conditions	467 356 (29.2)	152 129 (32.9)	315 227 (27.8)***
≥4 mental health conditions	847 268 (53.0)	243 715 (52.7)	603 553 (53.1)***
0 physical health condition	682 348 (42.7)	215 515 (46.6)	466 833 (41.1)***
1 physical health condition	396 692 (24.8)	117 338 (25.4)	279 354 (24.6)***
≥2 physical health conditions	519 527 (32.5)	129 878 (28.1)	389 649 (34.3)***
Urban–rural classification, *n* (%)			
Urban	1 306 985 (81.8)	375 161 (81.1)	931 824 (82.0)***
Rural	268 680 (16.8)	86 269 (18.6)	182 411 (16.1)***
Unknown	22 902 (1.4)	1 301 (0.3)	21 601 (1.9)***
Year, *n* (%)			
2003–2007	482 694 (30.2)	133 282 (28.8)	349 412 (30.8)***
2008–2011	401 246 (25.1)	113 557 (24.5)	287 689 (25.3)***
2012–2015	416 335 (26.0)	121 941 (26.4)	294 394 (25.9)***
2016–2019	298 292 (18.7)	93 951 (20.3)	204 341 (18.0)***
Discharge type, *n* (%)			
Irregular	139 208 (8.7)	91 159 (19.7)	48 049 (4.2)***
Regular	1 459 359 (91.3)	371 572 (80.3)	1 087 787 (95.8)***
Length of stay			
Days, median (IQR)	8 (21)	6.0 (8)	37 (57)

IQR, interquartile range.

*** *P* < 0.001, comparison between residential and acute in-patient.

With regards to mortality rates from the two settings, we found that the overall crude suicide, external-cause, and all-cause mortality rates at 90-day follow-up from acute in-patient settings were 582, 1419 and 3720 per 100 000 person-years, respectively. In comparison, the overall residential setting rates were substantially lower: 198, 1029 and 2371 per 100 000 person-years, respectively. Most deaths from external causes were related to poisoning (38%) or suicide (38%) with the remaining for other reasons such as motor vehicle accident, falls or suffocation.

### Survival analysis

Regardless of setting, patients were at greater risk of death if they left treatment after an irregular versus a regular discharge (Table 2). Most striking, among patients leaving a residential setting, the risk for suicide in the first 30 days was more than three times greater when comparing irregular versus regular discharge (adjusted HR = 3.41, 95% CI 2.21–5.25). In addition, among patients leaving an acute in-patient stay, the risk for suicide in the first 30 days of discharge was 55% greater when comparing patients with an irregular versus a regular discharge (adjusted HR = 1.55, 95% CI 1.11–2.16). The risk of mortality with an irregular discharge remained high in both settings for secondary outcomes.

**Table 2:** Mortality risk among patients discharged from acute in-patient or residential mental health treatment settings, Department of Veterans Affairs, 2003–2018^a^

	Suicide		External-cause mortality		All-cause mortality	
	Rate per 100 000 person-years	Adjusted HR (95% CI)	Rate per 100 000 person-years	Adjusted HR (95%CI)	Rate per 100 000 person-years	Adjusted HR (95% CI)
*Follow-up period censored at 30-days post-discharge*						
Acute in-patient mental health setting						
Irregular	1186	1.55 (1.11–2.16)	3679	1.75 (1.44–2.11)	5959	1.47 (1.26–1.7)
Regular	834	Ref	1925	Ref	4520	Ref
Residential mental health setting						
Irregular	604	3.41 (2.21–5.25)	2385	1.83 (1.49–2.23)	4137	1.51 (1.31–1.75)
Regular	188	Ref	1126	Ref	2744	Ref
*Follow-up period censored at 90-days post-discharge*						
Acute in-patient mental health setting						
Irregular	601	1.20 (0.9–1.59)	2143	1.40 (1.20–1.63)	4274	1.29 (1.16–1.44)
Regular	546	Ref	1390	Ref	3698	Ref
Residential mental health setting						
Irregular	335	2.05 (1.50–2.80)	1647	1.58 (1.37–1.81)	3072	1.38 (1.25–1.52)
Regular	168	Ref	889	Ref	2214	Ref
*Follow-up period censored at 180-days post-discharge*						
Acute in-patient mental health setting						
Irregular	503	1.22 (0.97–1.55)	1745	1.34 (1.18–1.52)	3738	1.21 (1.11–1.32)
Regular	428	Ref	1153	Ref	3386	Ref
Residential mental health setting						
Irregular	238	1.56 (1.20–2.02)	1332	1.47 (1.32–1.65)	2756	1.28 (1.19–1.38)
Regular	155	Ref	792	Ref	2146	Ref

HR, hazard ratios; Ref, reference.

a. Adjusted hazard ratios obtained from models that account for age, gender, race, marital status, number of prior mental health and physical health conditions, homelessness, rurality, primary discharge diagnosis and year of admission. Separate models were run for acute and residential stays.

We observed similar trends in the relationship between irregular discharge and mortality outcomes when we censored at 90 and 30 days, although the effect was slightly less pronounced in the first 30 days. For example at 90 dats, in the residential setting, patients were now at double the risk for suicide after an irregular discharge as compared with those with a regular discharge (adjusted HR = 2.05, 95% CI 1.50–2.80). We also observed in the acute in-patient setting that irregular discharge was no longer predictive of death by suicide (adjusted HR = 1.20, 95% CI 0.9–1.59).

Censoring follow-up time at 180 days, we observed that patients with an irregular discharge continued to be at higher risk for death compared with patients with a regular discharge (HR 1.2 to 1.6). These trends held true both for acute in-patient as well as residential settings. However, in both settings, the hazard was attenuated as follow-up time increased with the effect being less pronounced than was observed in the first 90 days.

**Fig. 1:**
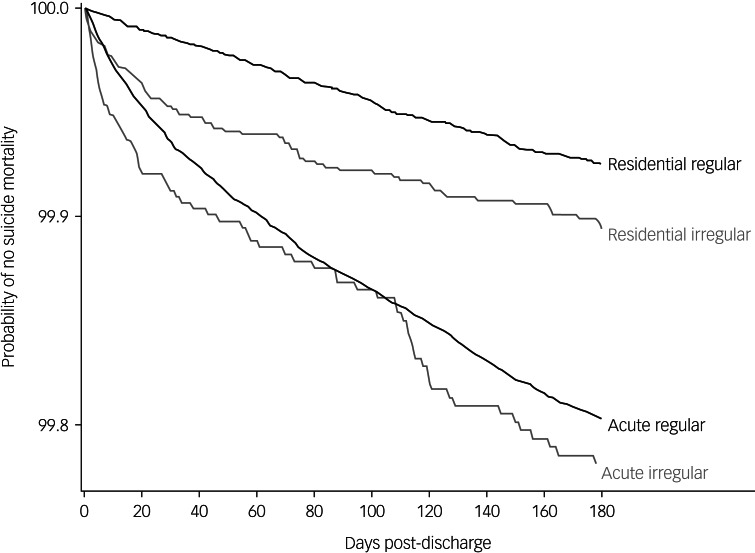
Probability of no suicide mortality within the first 180 days of irregular or regular discharge among acute in-patient and residential mental health treatment settings within the US Department of Veterans Affairs healthcare system, 2003–2018. The curves annotated residential refer to discharges from residential mental health treatment settings and the curves annotated acute refer to discharges from acute in-patient mental health settings.

**Fig. 2:**
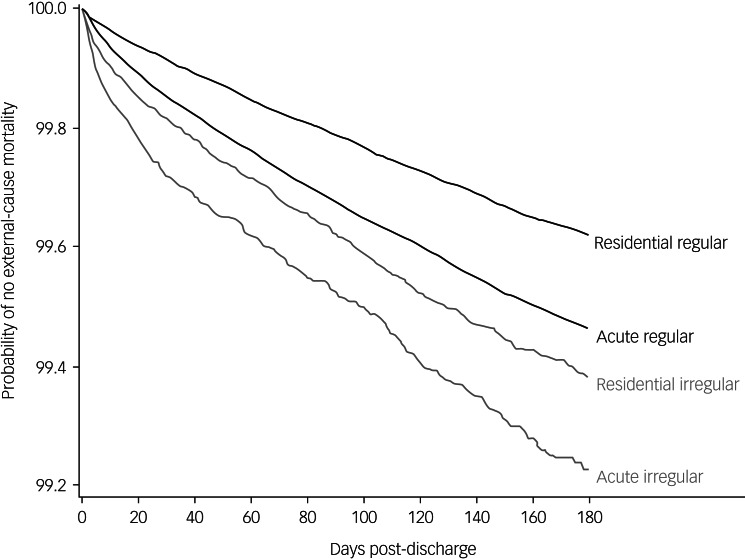
Probability of no external-cause mortality within the first 180 days of irregular or regular discharge among acute in-patient and residential mental health treatment settings within the USA Department of Veterans Affairs healthcare system, 2003–2018. The curves annotated residential refer to discharges from residential mental health treatment settings and the curves annotated acute refer to discharges from acute in-patient mental health settings.

Aligned with these findings, the survival curves for suicide (Fig. 1) and external-cause (Fig. 2) mortality indicated that the highest risk for death occurred in the first 30 days. Slopes in this early time frame were steep, especially for irregular discharges from the acute setting.

## Discussion

Irregular discharges from acute in-patient or residential settings are associated with increased risk for mortality in the first 6 months after discharge. Mortality risk is especially high among patients who are discharged from a residential setting. In fact, in our study, patients who left a residential stay after an irregular discharge experienced triple the adjusted risk for suicide within the first 30 days compared with patients with a regular discharge. Mental health providers should be acutely aware of the mortality risks associated with irregular discharge when developing discharge plans for this population

### Irregular discharge in residential settings and mortality outcomes

We found that irregular discharge from residential settings results in increased risk of suicide, external-cause and all-cause mortality for 6 months after discharge. Although our results generally align with the literature, it is difficult to directly compare our findings because prior studies generally focus on long-term rather than short-term mortality outcomes after residential treatment.^22,23^ For example, Harris et al found that among patients discharged from substance abuse residential rehabilitation programmes that early discharge (defined as the proportion of patients discharged within 1 week of admission) predicted 2-year all-cause mortality (odds ratio 1.49, *P* < 0.001).^22^ We also noted that patients with an irregular discharge were at 1.5 times greater risk for all-cause mortality within the first 30 days of a residential stay compared with those with a regular discharge. However, we found that risk attenuated over time with an observed increased risk of 28% in the first 180 days. Unlike Harris et al, our study includes a broader range of residential programmes and does not focus exclusively on programmes that treat SUD.^22^ Moreover, as with our study, Decker et al reported in a single-site study of patients admitted to a Veterans Affairs residential substance use treatment programme that failure to complete treatment was associated with a high risk of all-cause mortality in the first 5 years of discharge (HR = 2.3, 95% CI 1.1–4.9).^23^ The effect, however, was no longer significant after adjustment (HR = 2.1, 95% CI 0.9–4.6). Decker et al did not report on shorter-term risk for comparison.^23^ Finally, aligned with our results, in a 10-year follow-up study of 567 patients admitted with non-organic psychosis to New York hospitals, Craig et al noted that deaths because of unnatural causes tended to occur close after discharge from treatment.^24^ These authors, however, did not explore the role of irregular discharge on this risk.

### Irregular discharge in acute in-patient settings and mortality outcomes

Our observation that irregular discharge in the acute in-patient setting was predictive of all-cause, external-cause and 30-day suicide mortality somewhat aligns with trends reported in the literature.^2,3,25^ Riblet et al also found no relationship between irregular discharge and 1-year suicide risk following an acute in-patient mental health stay (HR = 1.16, 95% CI 0.96–1.40).^2^ It is hypothesised that the suicide risk associated with irregular discharge may be overshadowed by a pre-existing, high risk for suicide in this setting.^2^ Yet, unlike Riblet et al, we did find that patients with an irregular discharge had a 50% increased risk for suicide within the first 30 days of discharge from an acute in-patient stay, suggesting that time may influence risk. Our result also mirrors those of Kuo et al who reported that patients were nearly 50% more likely to die by suicide after an irregular versus regular discharge from an acute in-patient mental health stay (HR = 1.43, 95% CI 1.05–1.95).^3^ We are not aware of any studies that have looked at the relationship between irregular discharge in acute in-patient mental health settings and other mortality outcomes such as external-cause mortality. However, in a cohort study of 1.9 million patients with Veterans Affairs general medical admissions (2004–2008), Glasgow et al observed that an irregular discharge did not predict 30-day all-cause mortality (HR = 1.10, 95% CI 0.98–1.24), but did predict 60-day all-cause mortality (HR = 1.11, 95% CI 1.02–1.21).^25^ Our findings differ from Glasgow et al in that we found a significantly heightened risk for all-cause mortality at 30, 90 and 180 days. It is possible that the difference may be because of our focus on acute in-patient mental health stays (versus general medical stays). Glasgow et al also raised concerns that there was insufficient statistical power.^25^

We observed a significant increased risk for external- and all-cause mortality in the first 90 and 180 days after an acute in-patient discharge, but not for suicide. We may have simply lacked sufficient statistical power. Statistical power increases in survival analysis as the event rate increases. For example, only 11% of deaths in the first 30 days were attributable to suicide. The point estimates were also similar across outcomes and irregular discharge was always associated with increased risk. Conversely, it is possible that the risk for these outcomes may diminish at differential rates following irregular discharge from acute settings.

We highlight that the mortality rates were uniformly high after an acute in-patient or residential stay regardless of discharge type. This aligns with current trends in the literature,^9,10,26,27^ although there has been limited study of mortality rates following residential stays.^22,23^ For example, Valenstein et al also reported a rate of 568 suicide deaths per 100 000 person-years in the first 12 weeks after a psychiatric hospital stay among Veterans Affairs patients diagnosed with depression.^27^ Although Katz et al also observed high rates of all-cause mortality in the first 90 days after a Veterans Affairs mental health discharge, the authors cited higher rates than were reported in our study (4490 versus 3720 per 100 000 person-years).^26^ We speculate that this may reflect differences in study years or definitions used to index admissions, setting or discharge status.^26^

Finally, it is important to note that the crude suicide rates reported in our study were 20 to 50 times greater than global age-standardised suicide rates.^10^ Suicide and external-cause mortality rates were also higher among discharges from acute in-patient versus residential stays. This is not surprising as residential programmes typically care for a less acute population whose needs do not reach a level of requiring acute in-patient treatment.^7,8^

### Factors contributing to mortality risk with irregular discharge from mental health settings

Several factors may explain why patients are at greater risk for death after an irregular discharge from mental health treatment settings. Prior research has found that some of the key drivers of irregular discharge may include symptom worsening (such as undertreated withdrawal, uncontrolled pain),^28^ perceived stigma from staff^29^ and poor therapeutic environment (for example unit restrictions serve as a trigger for patients with prior incarceration).^29^ Incomplete treatment at the time of discharge may heighten the risk for symptom worsening.^30^ This may be especially true for patients with SUD who may experience withdrawal symptoms that drive them to abruptly leave the programme and, potentially, relapse.^30^ Providers’ beliefs about the degree of treatment that should be provided to patients who leave with an irregular discharge may also result in patients leaving without treatment (such as medication supply or scheduled follow-up care).^4,30^ Related to this concern, there may also be breakdowns in therapeutic alliance between providers and patients at the time of an irregular discharge. For example, patients may perceive that their needs were not addressed or feel generally alienated from providers because of stigma.^30^ These factors may put patients at a higher risk for adverse outcomes such as suicide because they are then isolated from necessary treatment and related out-patient supports. Aligned with these concerns, we found that the risk for suicide was particularly high in the first 30 days post-discharge (HR = 3.41).

In a root-cause analysis study of suicides following residential treatment, Riblet et al also noted that factors contributing to post-discharge suicide risk included precipitous discharges because of programme violations, inadequate treatment and insufficient processes to address irregular discharges, assess suicide risk or arrange follow-up care.^4^ In residential settings, the length of participation in the programme may also predict the likelihood of engagement in treatment after discharge. A study of 367 Canadian adults who completed abstinence-based residential addiction treatment reported that for each additional day the patient remained in the residential programme, there was a 2% increase in the likelihood of participating in aftercare.^31^

### Strategies to improve mortality outcomes after irregular discharge from mental health settings

Because irregular discharges from acute in-patient and residential stays are associated with higher risk for death after discharge, it is imperative that future research focuses on identifying effective interventions to prevent irregular discharge and related adverse outcomes. To date, a few studies have evaluated various strategies to prevent irregular discharge, although they have primarily focused on patients with SUD.^30,32,33^ For example, using retrospective data from over 35 000 hospital-related treatment encounters for SUD, Thompson et al found that compared with usual care, a substance use intervention team (SUIT) was associated with a significantly greater probability that patients would leave with a regular discharge (HR = 1.16, 95% CI 1.03–1.3).^32^ The SUIT intervention focuses on harm reduction and includes interventions such as medication initiation, motivational interviewing, treatment education and processes to facilitate post-discharge care.^32^ In a study of veterans discharged from an in-patient mental health recovery and rehabilitation programme, Decker et al also found that small group, patient-centred therapy interventions were associated with significant improvements in successful treatment completion and better treatment retention.^33^

Together, these findings suggest that successful approaches to address irregular discharge (and related adverse outcomes) may include adequate treatment of symptoms during admission and contingency plans to mitigate harms post-discharge and promote treatment engagement.^30,34^ Accordingly, the Veterans Affairs has implemented policies to ensure that patients with irregular discharge are offered an appointment within 24 h as well as an appointment within 7 days after discharge.^35^ Our findings, however, suggest that it may be necessary to promote engagement in treatment for a substantially longer period of time. Understanding the factors that contribute to irregular discharge is essential to intervention development, especially as contextual factors such as poor programme atmosphere, negative biases on the part of providers, and breakdowns in therapeutic alliance may contribute to irregular discharges and inadequate follow-up care.^29,30^ Although our study controlled for a broad array of confounders, including prior mental and physical health conditions and homelessness, unmeasured confounding variables may have influenced our findings. Interventions should be tailored to address the unique drivers of irregular discharge (and related harms) as there is likely no one-size fits all solution.

### Study strengths and limitations

A clear strength of the study is use of a large and lengthy cohort derived from the largest integrated healthcare system in the USA. This is the first study to report on a broad range of mortality outcomes among patients discharged from acute in-patient and residential settings after irregular discharge. We also were able to adjust for patient-level factors highly predictive of the outcome. There are several limitations to our analysis. First, we relied entirely on administrative data to identify demographic and clinical information. We cannot infer causality because patients were not randomly assigned to conditions. Second, our study reports on members of the US veteran population who accessed Veterans Affairs care and most patients were men, thus limiting the generalisability of our findings. Compared with the general US population (and veterans who do not access Veterans Affairs care), veterans who access Veterans Affairs care tend to be older, have more health comorbidities, have higher rates of

homelessness, and are socioeconomically disadvantaged.^12^ These factors as well as military experience may contribute to irregular discharge and post-discharge mortality. We also did not consider the role of non-Veterans Affairs mental health admissions on mortality outcomes because these care processes are outside of direct control of the Veterans Affairs. Finally, the survival curves point to clear differences in effect sizes based on treatment setting, highlighting an interaction between setting, discharge type and outcome. Because the analyses are crude, we cannot draw any formal conclusions.

In summary, we found that regardless of setting, the risk for death after an irregular discharge (versus a regular discharge) is the greatest within the first 30 days of discharge. Overall, our results emphasise that future research should focus on identifying effective interventions to address irregular discharges in acute in-patient and residential settings. These strategies should target related adverse health outcomes.

## Data Availability

The data sources used in this study cannot be made available to the general public because these data include sensitive information. The data source for this study includes the Veterans Affairs Corporate Data Warehouse (CDW). The Veterans Affairs CDW is comprised of electronic medical record data that is compiled from individual Veterans Affairs facilities and is described here: http://www.hsrd.research.va.gov/for_researchers/vinci/cdw.cfm. The data are stored on geographically dispersed server farms. In order to access the CDW, researchers would need to have an employment relationship with the Veterans Affairs. Then, after local institutional review board approval, a request for access to the data must be submitted to Veterans Affairs National Data Systems using the Data Access Request Tracker. The data-sets are then built and analysed within a secure virtual project workspaces housed within the Veterans Affairs Informatics and Computing Infrastructure environment. Researchers with direct access to the Veterans Affairs network can obtain further descriptions of CDW data located here: http://vaww.virec.research.va.gov/.
